# Cost-effective fabrication of photopolymer molds with multi-level microstructures for PDMS microfluidic device manufacture†

**DOI:** 10.1039/c9ra07955f

**Published:** 2020-01-23

**Authors:** Carol M. Olmos, Ana Peñaherrera, Gustavo Rosero, Karla Vizuete, Darío Ruarte, Marie Follo, Andrea Vaca, Carlos R. Arroyo, Alexis Debut, Luis Cumbal, Maximiliano S. Pérez, Betiana Lerner, Roland Mertelsmann

**Affiliations:** Universidad Tecnológica Nacional (UTN), Facultad Regional Haedo Haedo Buenos Aires E 1706 Argentina belerner@fiu.edu; Department of Medicine I, Medical Center – University of Freiburg, Faculty of Medicine, University of Freiburg Germany roland.mertelsmann@uniklinik-freiburg.de; Universidad de las Fuerzas Armadas ESPE, Centro de Nanociencia y Nanotecnología P. O. Box 171-5-231B Sangolqui Ecuador; Department of Electrical and Computer Engineering, Florida International University Miami Florida 33174 USA

## Abstract

This paper describes a methodology of photopolymer mold fabrication with multi-level microstructures for polydimethylsiloxane (PDMS) microfluidic device manufacture. Multi-level microstructures can be performed by varying UVA exposure time and channel width. Scanning Electron Microscopy (SEM), Atomic Force Microscopy (AFM) and profilometry techniques have been employed to characterize the molds. Multiple molds with multi-level microstructures can be formed in a unique piece. Overall height/depth of the structures reaches up to 677 μm and a minimum of 21 μm. The method provides several advantages such as reduction of fabrication time, multiple structures with diverse topologies, a great variety of depth and height in a single mold and low cost of fabrication. The effectiveness of multi-level microstructure fabrication was evaluated by constructing PDMS microfluidic devices for cell culture and proliferation.

## Abstract 2

This paper describes a methodology of photopolymer mold fabrication with multi-level microstructures for polydimethylsiloxane (PDMS) microfluidic device manufacture.

## Graphical abstract

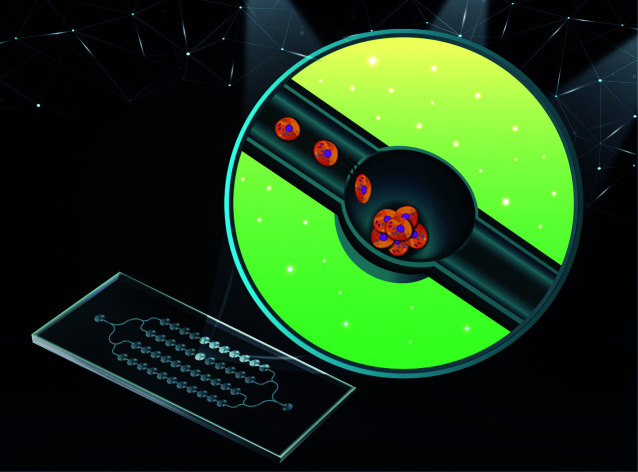

## Introduction

Several methods have been developed over the last few years to fabricate multi-level microfluidic structures in order to enhance the performance and/or to increase the capabilities of a variety of microfluidic devices.^1^ Within the methods are membrane sandwich,^2^ reaction-diffusion,^3^ multilayer processes,^4^ diffuse UV exposure erosion,^5^ etching,^6^ laser fabrication,^7^ photoresist reflow,^8^ micromilling,^9^ 3D-printing,^10^ electroplating,^11^ photolithography,^12^ and liquid molding.^13^ Recent applications of multi-level microstructures have been reported for colloidal particle separation,^14^ separation and extraction of microparticles,^15^ neuron culture,^12^ microfluidic mixers,^5^ cancer research applications,^16^ chemical processing,^17^ and to produce microvascular networks for *in vitro* laboratory systems,^11^ among others. In this sense, the development of novel and cost-effective methods for the fabrication of microfluidic devices with multi-level structures has recently gained considerable attention in the scientific and industrial communities.

In cell biology, the multi-level microstructures present great potential for cells culture and proliferation. In regard, suspension cells culturing within microfluidic devices is challenging because simply renewing the medium can lead to accidental cell loss without a trapping mechanism.^18,19^ Yue *et al.* reported the fabrication of multi-level channels for cell quantification and cell culture by using a screen printing method.^20^ Moreover, microchannels with trapezoidal cross-section have been reported as a retention mechanism that enabled the capture and suspension culture of mammalian cells through inertial microfluidics.^21,22^ Besides, a microenvironment mimetic microfluidic device has been fabricated with a microchannel with a micro-well branched structure to simulate tumor microenvironment interactions.^23^ At present, microfabricated spherical chambers have been used as a micro pocket culture system to retain breast cancer cells aggregates.^19^ Furthermore, long term live-cell imaging of immune cells is demanding due to their non-adherent nature.^24^ Therefore, several strategies have been used to try and overcome these problems, such as micro droplet encapsulation,^25^ optical trapping,^26^ and cell isolation arrays.^27^ Thus, the fabrication of molds with multi-level microstructures for the PDMS microdevice fabrication presents a useful potential for biological applications.^18,20,28,29^

Recently, we reported the manufacture of PDMS microfluidic devices by employing a flexographic photopolymer as mold.^30,31^ Advantages related to resolution, mold size, aspect ratio, roughness, availability, scalability and costs were demonstrated.^30^ It is possible to obtain minimum channel widths lower than non-traditional techniques such as CO_2_ laser ablation,^32^ building blocks,^33^ laser ablation,^34^ laser swelling,^35^ semi-contact writing,^36^ 3D printing,^37,38^ WAX mold.^39^ In regard to roughness, the Fmold present smoother structures in contrast to techniques such as CO_2_ laser ablation,^32^ laser ablation^34^ and 3D printing.^37,38^ On the other hand, between the methodologies to obtain inexpensive devices are the microfluidic paper-based, PMMA based and F-mold based.^30,40–42^ Thus, it is possible to obtain devices cheapest than the fabricated by using etching (FIB),^43^ lytography (photoresins),^43–45^ electron beam lithography^46^ traditional techniques. Finally, the reported methodology allowed obtaining molds with different topologies and channeling dimensions (length, width, and height). Furthermore, large molds with dimensions of 1270 × 2062 mm^2^, structure heights ranging from 53 to 1500 μm, can be manufactured keeping the same resolutions (10 microns) that are obtained in small standard wafer size molds.^31^

In this work, we have developed an unconventional mold fabrication methodology with multi-level positive and negative structures by using the flexographic photopolymer. Multi-level structures were performed by varying UVA exposure and channel width. The resulting molds were characterized by Scanning Electron Microscopy (SEM), Atomic Force Microscopy (AFM) and profilometry techniques. Furthermore, we tested the multi-level structures of PDMS microfluidic devices to demonstrate its biological viability and functionality by monitoring Jurkat cell proliferation.

## Experimental

### Photopolymer mold fabrication

#### Multi-level microstructure fabrication by varying UVA exposure time and channel width

##### Multi-level microstructures fabrication by varying UVA exposure time

**Fig. 1:**
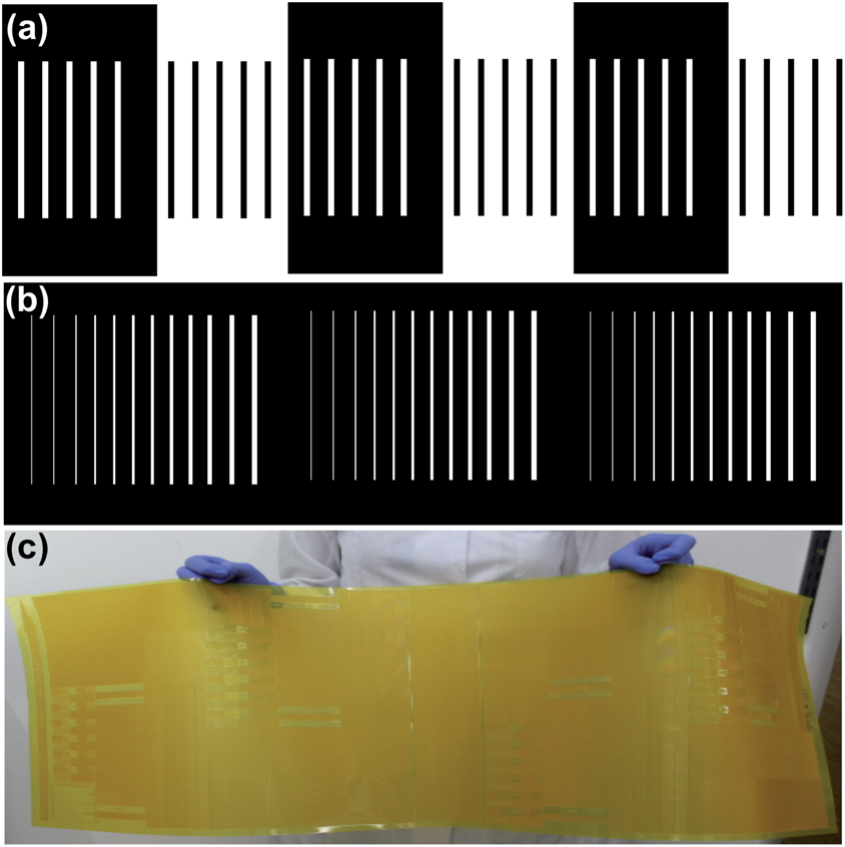
Photopolymer mold fabrication. (a) Microchannels network designed for the multi-level microstructures fabrication by varying UVA exposure time, (b) microchannels network designed for the multi-level microstructure fabrication by varying channel width and UVA exposure time (c) multiple multi-level structures in a single photopolymer mold. The designs of the mold exhibit in the figure (a) and (b) were performed on the same photopolymer plate.

Photopolymer Flexcel NX and Thermal Imaging Layer (TIL) supplied by Eastman Kodak^47^ were used in the fabrication of the molds. Mold fabrication process consists of various steps: (1) microchannels network was designed with a layout editor software^48^ and transferred to the TIL with an infrared laser source of 2400 ppi, (2) the TIL was laminated onto the unexposed photopolymer plate, (3) the photopolymer plate was exposed to UVA light at 0.45 J on the back side during 10 s, (4) a part of the photopolymer was covered with a mask plate on the back side, (5) the photopolymer plate was exposed to UVA light at 0.45 J on the back side for 20 s, (6) the steps 4 and 5 were repeated one time, (7) the front side was exposed to UVA light at 19 J for 360 s, after the TIL was removed, (8) the photopolymer plate was washed with solvent PROSOL N-1 (supplied by Eastman Kodak) at 360 mm min^−1^ and dried in an oven during 30 min at 50 °C, (9) the photopolymer plate was exposed to UVC light at 10 J for 17 min and UVA light at 4 J for 2 min applied on the front side. The steps 2 to 6 are shown in Fig. 1. The design consists of adjacent rectangular structures with a width of 1000 μm (Fig. 1a).

The mold fabrication with multi-level structures was performed using the procedure described previously. Three equal sections were designed. Each section presents adjacent rectangular structures with a width of 50 μm, 100 μm, 150 μm, 200 μm, 250 μm, 300 μm, 350 μm, 400 μm, 450 μm, 500 μm, 550 μm and 600 μm. UVA exposition at 0.45 J on the back-side during 10, 30 and 50 s were performed on Section 1, 2 and 3, respectively (Fig. 1b).

##### Mold characterization

The morphological characterization of the molds was performed on a Field Emission Gun Scanning Electron Microscope (TESCAN FEG SEM MIRA3). Previous to the analysis, the molds were metalized with approximately 20 nm gold layer with a sputtering evaporator (Quorum Q150R ES). SEM measurements were carried out at 7 kV and the quantitative measurements were made with the MIRA TC software version 4.2.24.0. Profilometry measurements were performed on a Dektak XT profilometer from Bruker. Analyses were carried out with Vision 64 software. Linear scans were performed with a 25 μm radius tip, at a scan speed of approximately 90 μm s^−1^ a sampling rate of 0.01 Hz mm^−1^. Before characterization, the molds were blown with nitrogen gas to remove dust and then were ultrasonically cleaned in ethanol (70% v/v) for ten minutes (this step was repeated 5 times). Afterward, the molds were dried in an oven at 40 °C for 1 hour. AFM images were acquired in ScanAsyst mode at ambient conditions by using a cantilever of spring constant at 0.71 N m^−1^. The average roughness (*R*_a_) parameter was determined by applying the Nanoscope Analysis 1.8 software to multiple images taken at random positions in scan areas of 50 × 50 μm^2^. AFM images reported in this work were reproducible over at least five points on the sample surface.

#### Application

##### Microfluidic device fabrication

In a first step the mold was manufactured. The microchannel network was designed using Layout Editor Software and then, it was transferred to the Thermal Imaging Layer (TIL). Subsequently, the female photopolymer mold (Fmold) fabrication was performed as described in the previous section (Multi-level microstructures fabrication by varying UVA exposure time). However, the back-side of the photopolymer plate just was exposed one time during 10 seconds. The design consists of four lines with eleven wells in series within each one. The positions of the wells are denoted as L_i__W_j_, where L_i_ corresponds to the line number and W_j_ correspond to the well number (Fig. 5a). In the next step, two PDMS replicas (PDMS-floor replica and PDMS replica with design) were manufactured in order to fabricate the microfluidic device. Briefly, a mixture of epoxy resin and curing agent (Cristal-Tack, Novarchem – Argentina) was poured onto the female mold to replicate the design in high relief. After curing, the epoxy resin mold (ERmold) was peeled off from the Fmold to form the male mold. Subsequently, a mixture of PDMS and curing agent in a 10 : 1 weight ratio (Sylgard 184 Silicone Elastomer Kit) was poured onto the ERmold and cured in an oven at 40 °C overnight. For the PDMS-floor replica (replica without design), 3 grams of PDMS-curing agent mixture was poured on a glass slide and cured. Finally, the PDMS-floor replica and the PDMS replica with the design were exposed to oxygen plasma produced by BD-10A High-frequency generator (Electro-Technic Products, USA) to bond them irreversibly.

##### Cell culturing

Microfluidic devices were flushed with ethanol 70%, NaOH (0.5 M) during 30 minutes and ultrapure water before seeding cells. The leukemic cell line Jurkat (ACC-282, Leibniz Institute German Collection of Microorganisms and Cell Cultures, DSMZ) was cultivated using Roswell Park Memorial Institute (RPMI) 1640 medium (Gibco) supplemented with 10% fetal bovine serum (FBS) and 1% Pen Strep (10 000 μg ml^−1^ penicillin, 10 000 μg ml^−1^ streptomycin; Gibco). Jurkat cells were incubated at 37 °C in a humidified atmosphere containing 5% CO_2_ and passaged to a new flask containing fresh medium every three days. A volume of 65 μl of Jurkat cell suspension at a concentration of 3.33 × 10^5^ cells per ml^−1^ was injected into each line of the microfluidic device using an A22 syringe pump (ADOX, Argentina) at flow rate of 26 μl min^−1^ during 3 minutes. The syringe pump was rotated 90° to prevent cell sedimentation along the syringe lateral wall. Medium was renewed at day 3 using a flow rate of 2 μl min^−1^ during 35 minutes for each line of the microfluidic device. Cell concentration in medium extracted from the outlets was measured with TC10™ Automated Cell Counter Bio-Rad (Lighthouse Core Facility, Germany). Jurkat cells were microscopically monitored and images were acquired at days 1 to 5 with an inverted Zeiss Observer microscope (Lighthouse Core Facility, Germany) using the EC Plan-Neofluar 2.5×/0.075 and 10×/0.3 objectives. In order to prevent evaporation, the microfluidic device was placed inside a Petri dish supplied with water reservoirs. The Petri dish was taken out from the incubator to transport the microfluidic device to the microscope. Image sequences of Jurkat cells after being seeded were obtained with an Olympus Scan^R microscope (Lighthouse Core Facility, Germany) and are presented in ESI Video S1.†

##### Image analysis

In order to estimate cell proliferation, the area occupied by the suspension cells was measured using a macro written in FIJI Image J. The day of seeding was considered as day 1, while the day after Jurkat cells settled was considered as day 2. The area of the whole image was considered to be 100% and the percentage of area occupied by the cells was reported. Seven wells were monitored microscopically and images were acquired daily.

##### Identification of dead cells

For detecting cell death events, culture medium was supplemented with 0.25 μg ml^−1^ of Propidium Iodide (PI)^49^ and it was flushed into the microchannels at day 5. PI is a DNA intercalating dye which does not pass through the plasma membrane of viable cells as described by Zaretsky *et al.*^50^ Images were acquired with the inverted Zeiss Observer microscope using the PI filter set. To state the percentage of dead cells, the total cell area on the bright field image was considered to be 100% whereas the area of cells which were PI-positive was reported as the area of dead cells. This measurement allowed determining the biological viability of the multilevel structures of the device.

## Results and discussion

The molds fabrication of the present study was carried out by using a photopolymerization process.^31^ The photopolymerization of the plate by UVA exposure on the back-side is the step that controls the height/depth of the structures and to support the anchoring of fine details.^31^ This step starts with a radical chain reaction of the reactive monomers that react with other monomers and the polymer binder and form a crosslinked network. As long as the UVA exposure is switched on the polymerization continues.^31^ In regard, in a previous work, an inverse relationship between UVA exposure time and structure height was demonstrated.^31^ In addition, the exposure on the front side is carried out with UVA light in order to form the structures on the polymer. After UVA exposure on the front side, the photopolymer plate is chemically and mechanically washed. Therefore, unexposed parts of polymer material are removed and structures are formed. In this sense, the proposed methodology in this work enables the fabrication of multi-level structures on a single mold by varying the UVA exposure time and/or the channel width. In the next sections, the variation of each one of these factors as well as the result of modifying both will be described. In addition, an application of multi-level microfluidic device will be shown.

### Multi-level microstructures fabrication by varying UVA exposure time

**Fig. 2:**
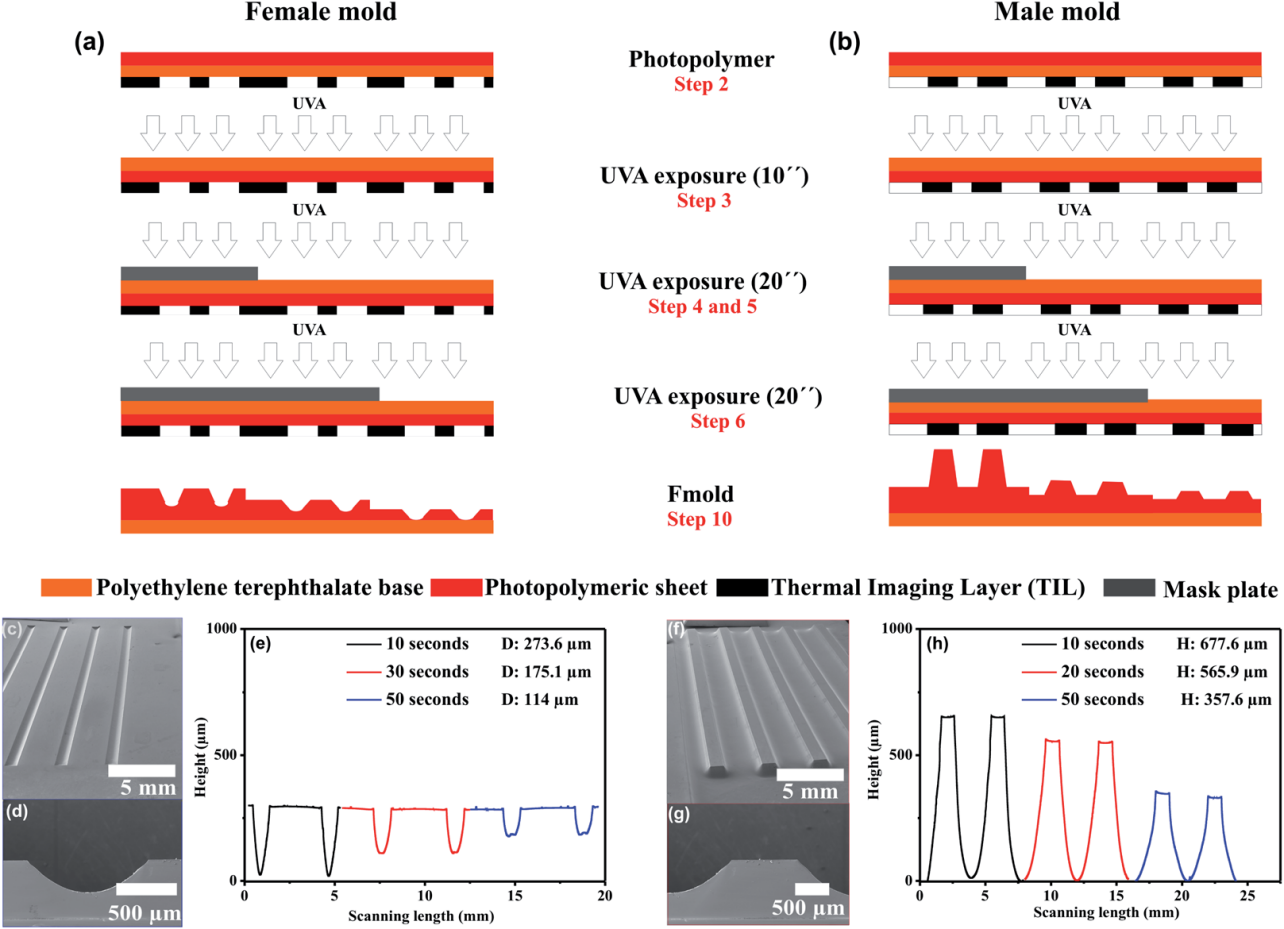
Multi-level structures on female and male molds. (a) and (b) Schematic drawing of principal steps to fabricate multilevel microchannels, (c) and (f) SEM images of top-view female and male molds, (d) and (g) cross-section of female and male molds, (e) and (h) depth/height channel measurements recorded by profilometry. Using the L-edit software, channel width was drawn of 1000 μm. Three section of the photopolymer plate were exposed in total to UVA light at 0.45 J on the back side during 10 s, 30 s and 50 s, respectively. This corresponds with the three UVA exposure steps of 10 s, 20 s and 20 s. D: Depth channel, H: height channel.

Fig. 2a and b show the principal steps to fabricate molds with male and female multi-level microchannels. As it can be seen in the figure the photopolymer mold was exposed multiple times. Representative SEM images of manufactured female and male molds are shown in Fig. 2c–g. The height and depth channel measurements obtained by profilometry are included in Fig. 2e and h. The results demonstrate that height/depth of the structures varies according to UVA exposure time. For UVA exposure of 10, 30 and 50 were obtained positive structures (male mold) with a height of 677.6, 565.9 and 357.6 μm, respectively. On the same photopolymer plate, negative structures (female mold) were performed with deep of 273.6, 175.1 and 114 μm, respectively. From the results, it can be seen that an inverse relationship between UVA exposure time and height/depth structure exists. Furthermore, it can be observed that female molds show structures with lower thickness in comparison with male molds, this is due overlapping of the sidewalls (Fig. 2d). For male mold, the heights of the structures are dependent on the spacing between individual structures have been reported in previous work.^31^ The results demonstrate that on a single photopolymer plate, it is possible to obtain uniform male/female molds with a great variety of height/depth dimensions on the microstructures by varying UVA exposure time (ESI Fig. S1†).

### Multi-level microstructure fabrication by varying UVA exposure time and channel width

**Fig. 3:**
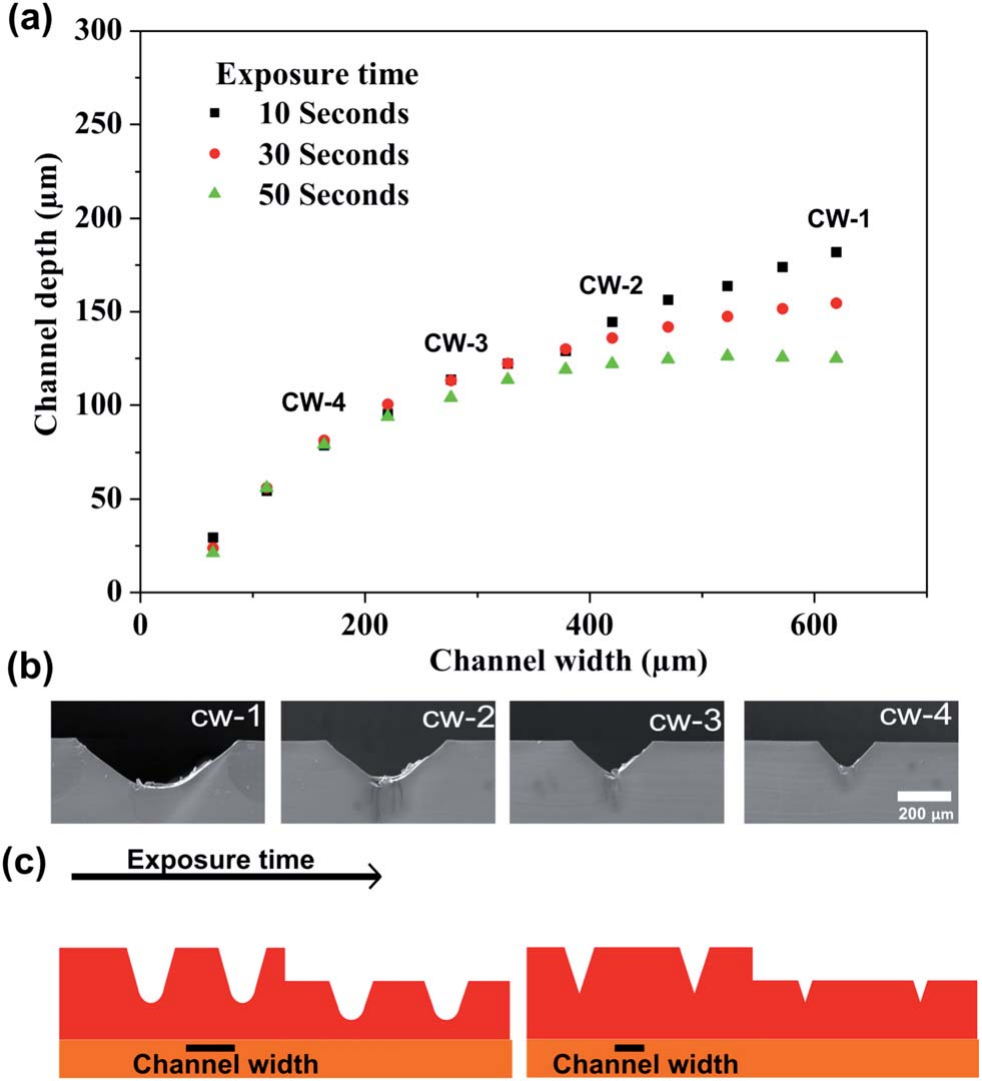
(a) Channel depth as a function of channel width and UVA exposure time, (b) cross section of channels obtained by UVA exposure on the back side by 10 s. (c) Schematic representation of depth of the structures obtained by varying the channel width and exposure time. Depth and width measurements were determined by profilometry technique (*n* = 3). cw: channel width.

Fig. 3 shows the channel depth as a function of channel width and UVA exposure time. The procedure of the mold fabrication consisted of the variation of both parameters in a single photopolymer plate. The mask of the mold is shown in Fig. 1; adjacent linear structures with different channel widths were designed.

The photopolymer was exposed on the back-side by 10, 30 and 50 s, respectively. Depth measurements of the microstructures are exhibited in Fig. 3a which indicates that channel width between 50 μm and 300 μm presents similar depth under the three UVA exposure time applied. Similar behavior was observed when applying UVA exposure time of 30 s and 50 s and channel widths were lower than 400 μm. While channels width higher than 400 μm showed a considerable variation of the depth. The results indicate that the height of the structures with channel width lower than 400 μm was not dependent on the time exposure. This behavior is due to the trapezoidal shape that presents the structures; this characteristic shape generates an overlapping of sidewalls of the structures as shown in the schematic representation (Fig. 3c). SEM images show clearly the decreasing of the depth of the structures by effect of the overlapping of the sidewalls (Fig. 3b). Finally, the results indicate that the multi-level structures can be performed by varying channel width and UVA exposure time in a single step.

### Mold characterization

**Fig. 4:**
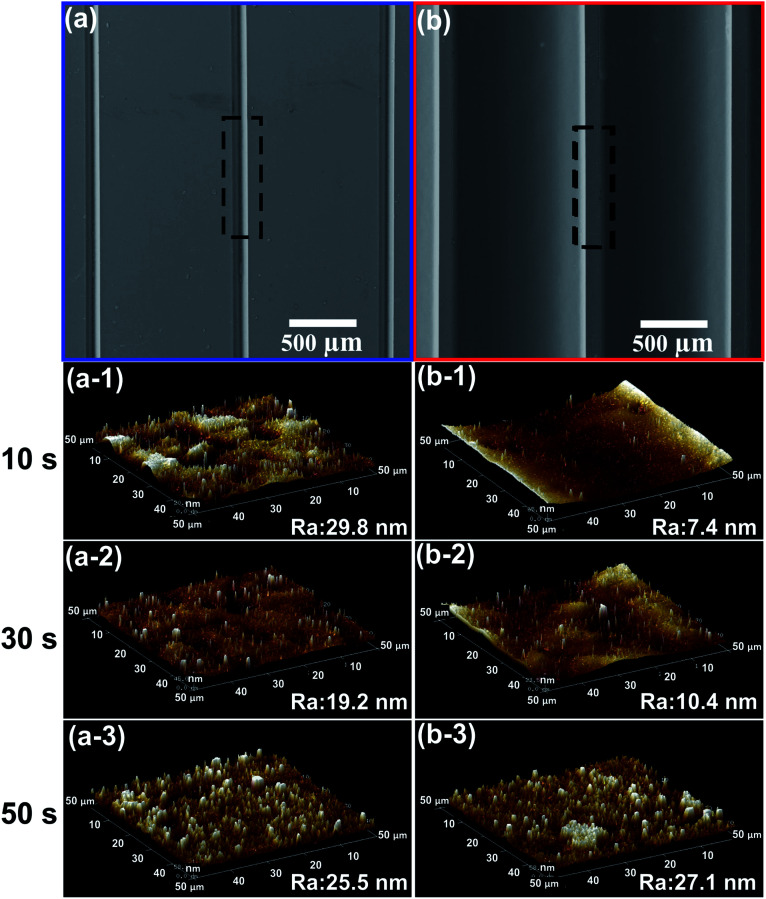
Surface morphology. Representative SEM images of top-view female (a) and male (b) molds, representative AFM images and *R*_a_ values obtained from the female molds (a1 to a3), representative AFM images and *R*_a_ values obtained from the male molds (b1 to b3). *R*_a_ represents the average roughness values. First step: UVA exposure time on back side: 10, 30 and 50 s, respectively. Exposure time on front side: 360 s, second step: UVA front exposure: 2 min, UVC front exposure: 17 min.

The effect of UVA exposure time on the surface morphology was studied by measuring the roughness of the photopolymer molds. Fig. 4 shows the average roughness values (*R*_a_) and representative SEM and AFM images recorded from the male and female molds. On the female mold, it can be observed a direct dependency between the UVA exposure time and the roughness. In contrast, the roughness of the male mold did not show any correlation with the UVA exposure time. Moreover, the roughness of male/female molds was lower than 29 nm with exposure time up to 50 s. In previous work, we reported an average roughness (*R*_a_) of photopolymer male molds, obtained from printing plate photopolymer Flexcel SRH. *R*_a_ values between 23 nm and 99 nm with exposure time lower than 48 s were obtained for the Flexcel SRH photopolymer. As rough surface could be a problem for imaging,^12^ it is important to obtain molds with the lowest possible roughness. In this sense, the Flexcel NX is the best choice for the fabrication of molds.

### Application

#### PDMS microdevice

**Fig. 5:**
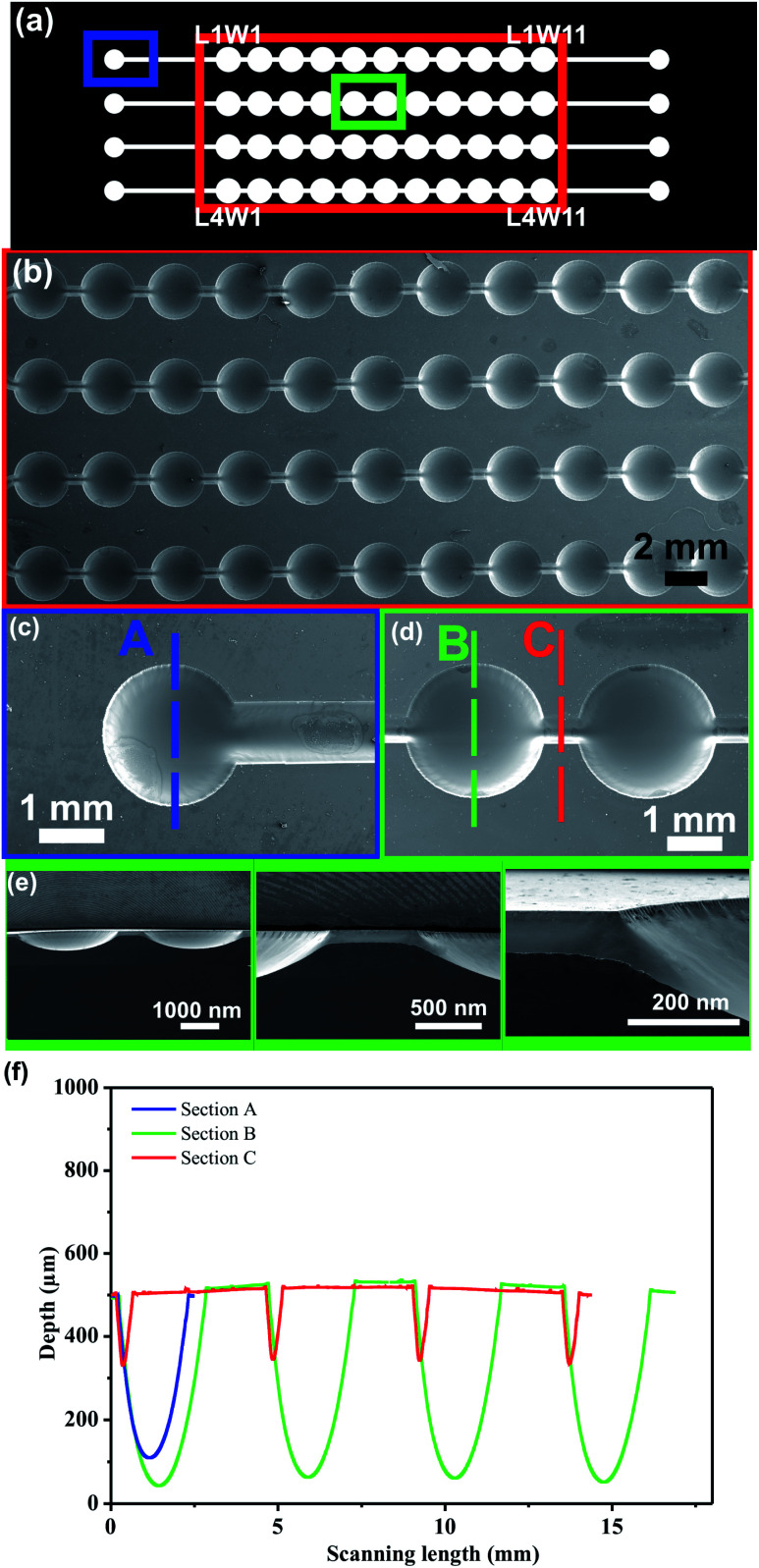
(a) Microchannels network of the microfluidic device (b) SEM image of the wells, (c) higher magnification SEM images of zone A, (d) higher magnification SEM images of zone B and C, (e) SEM images of the cross section of the mold (f) depth measurements recorded by profilometry. Photopolymer mold fabrication conditions: first step: UVA exposure time on back side: 10 s, exposure time on front side: 360 s, second step: UVA front exposure: 2 min, UVC front exposure: 17 min.

The effectiveness of multi-level microstructures fabrication was evaluated by constructing PDMS microfluidic devices for cells culture and proliferation. The multi-level microstructures fabrication was performed varying channel width in a female mold. As shown in Fig. 5, the design consists of an input and an output connected to four lines with eleven wells in series within each line. Fig. 5b–d show SEM images of the mold. The design presents circular and linear topologies. The channel width measurements were 2100 μm, 2600 μm and 530 μm in section C, A and B, respectively. The profilometry measurements show that depth dimensions are different in each section (Table 1). The results demonstrate proportional increases in channel depth as the channel width increments. Furthermore, it is observed that the results are in good agreement with the results obtained in the previous section.

**Table 1:** Dimensions resulting for section A, B and C^a^

Dimensions	Section		
	A	B	C
Width (μm)	2100 ± 0.01	2600 ± 0.01	530 ± 2.65
Depth (μm)	387 ± 3.23	468 ± 7.14	175 ± 0.01

a Depth and width measurements were determined by profilometry technique (*n* = 3).

#### Cell culture and proliferation in the multilevel microfluidic device

**Fig. 6:**
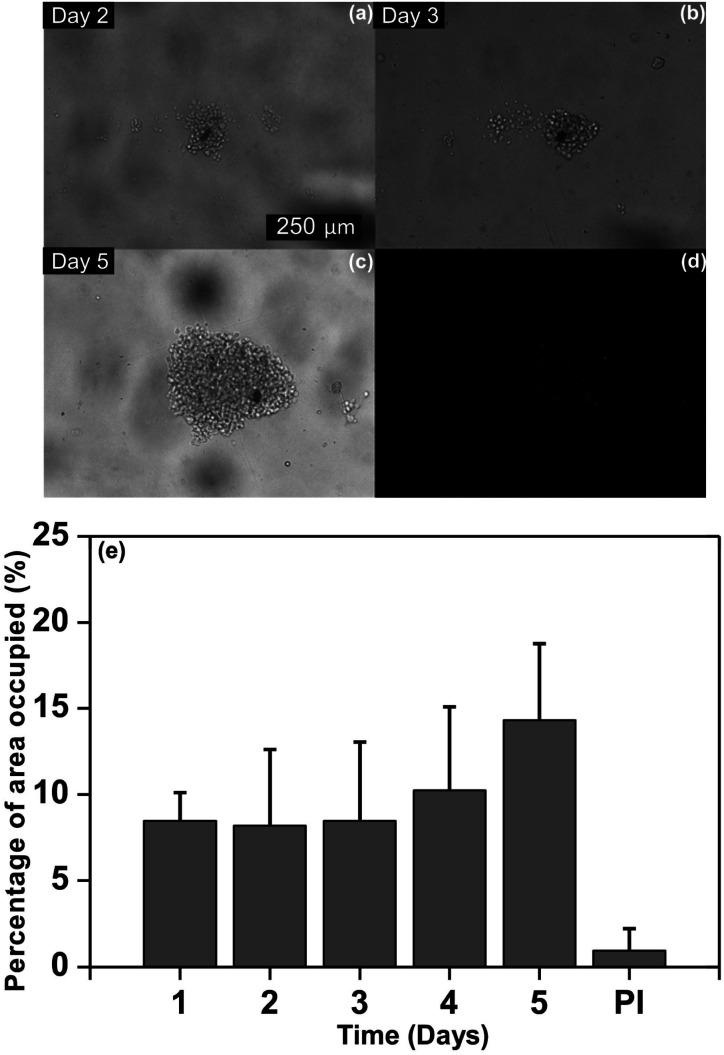
Proliferation of Jurkat cells in position L_4__W_1_ for 5 days. (a) day 2, (b) day 3, (c) day 5 and (d) detection of dead cells labeled with PI at day 5, (e) average of the area occupied by the Jurkat cells from day 1 to day 5. The last bar shows the percentage of area occupied detected for PI positive cells. The area occupied by the Jurkat cells was analyzed by Image J-FIJI. Experimental conditions: after seeding (day 1), Jurkat cells were imaged in the microfluidic device. At day 3 the medium was renewed using a flow rate of 2 μl min^−1^ during 35 minutes. Cellular death events were determined by end point death/live assay.

The fabrication of microdevices with multi-level microstructures presents useful potential for cell culture and proliferation.^20^ In this sense, Jurkat cells were cultured in the multilevel microfluidic device as a proof of principle for non-adherent cell capturing within wells for subsequent cell culture and proliferation. A volume of 65 μl of Jurkat cell suspension was injected into each line of the microfluidic device using. It was observed that the Jurkat cells were dispersed along the wells (Fig. 6d), subsequently, they settled by gravity and grouped at the deepest part of the wells. This behavior highlights the utility of the multilevel device as cells were immobilized at the bottom (zone B) whereas the upper zone permitted medium flow (zone C). In order to estimate the proliferation of Jurkat cells settled, the cells were microscopically examined over time and the area of the image occupied by them was measured. Fig. 6 shows the proliferation of Jurkat cells in position L_4__W_1_ during 5 days and Fig. S2 (ESI†) presents a collection of images of the 11 wells of the first line of the microfluidic device, after being seeded. Fig. 6a–c demonstrate that Jurkat cells proliferated. Moreover, Fig. 6e exhibit the proliferation of Jurkat cells by an increase in the average of the area occupied by the Jurkat cells from day 1 to day 5. The ESI Video 1† shows the capacity of the multilevel device to capture Jurkat cells. In addition, the medium was renewed at day 3 and cell concentration at outlets was under the detection limit of the automatic cell counter (5 × 10^4^ cells per ml^−1^), and no changes were observed by optical microscopy in the chambers, which indicated the absence of cell loss at a flow rate of 2 μl min^−1^.

The identification of dead cells was evaluated in order to determinate the viability of the multilevel structures on the microfluidic device for the cell culture and proliferation. To address this issue, the percentage of dead cells was quantified by image analysis through PI fluorescence following the labeling procedure assessed by Wlodkowic *et al.*^49^ and performed by Zaretsky *et al.*^50^ Furthermore, the area occupied by dead cells was measured by FIJI-Image J^51^ and it was related to the area covered by the cell cluster at day 5 as illustrated in Fig. 6. An average of the area occupied of 0.95% in the wells was determined which correspond with 5.9% of dead cells. By flow cytometry, the reported normal value of dead cells is less than 5%.^52^ While the values quantified by image analysis are between 5.6 ± 2% and 6.7 ± 2% under normal culture conditions.^49^ As less than an average of 10% of dead cells was determined, our multilevel device harbored Jurkat cells without detrimentally affecting their development. These results demonstrate that multilevel microstructures allow renewing the medium avoiding cell loss.

From the results, the multilevel microfluidic device showed the ability to harbor cells in order to monitor their proliferation over a 5 day time period. Moreover, the characteristics of the PDMS microdevice made possible the acquisition of images as the decreased thickness of the bottom PDMS replica (∼2 mm) which contains the microchannels design provides an appropriate working distance when using the 10× objective. Furthermore, the proposed microdevice design allowed harboring the cells at the deepest part of each well, allowing cells proliferation. These approaches allowed the characterization of suspension cell development due to the different levels make it possible to gently trap cell clusters within the wells while allowing the medium to be renewed. Fabrication of multilevel microstructures by traditional photolithography methods is expensive, requiring multiple lithography steps and repeated processing.^12^ In this sense, this work overcomes these challenges by using Fmolds.

## Conclusions

We successfully demonstrated the Fmold manufacture with multi-level channels by performing sequential back side UVA exposure time on the photopolymer and varying the channel width. Moreover, it was also showed that varying the channel width could control the thickness of the structures which represents a powerful feature as thickness could be customized according to the type of assays. Moreover, the method allows obtaining multiple molds with a great variety of dimensions and topologies in a single large area (1270 × 2062 mm^2^) reaching a minimum structure size of 10 μm and structures height ranging from 53 to 1500 μm. In addition, the molds can be commercially obtained at low cost and are worldwide available. Also, they can be used multiple times with the acquisition of reliable replicas without delamination as the mold and the structures designed comprise a unique piece. Besides, the mold is economically feasible, because the Flexcel technology can be commercially obtained at low cost. Finally, the results demonstrated that multi-level microfluidic device enabled medium renewal without detrimentally affecting cell proliferation. The findings suggest that the developed methodology to generate multilevel structures has great potential in different microfluidics fields, in particular, it presents an alternative for non-adherent cells culturing.

## Conflicts of interest

There are no conflicts to declare.

## Supplementary Material

RA-010-C9RA07955F-s001

RA-010-C9RA07955F-s002
