# Supplementary material for: Cost-effective fabrication of photopolymer molds with multi-level microstructures for PDMS microfluidic device manufacture
Source: RSC Adv. 2020 Jan 23;10(7):4071–9. doi: 10.1039/c9ra07955f (PMC9048755; doi:10.1039/c9ra07955f)
Supplement: RA-010-C9RA07955F-s001 [file RA-010-C9RA07955F-s001.pdf]

Electronic Supplementary Material (ESI) for RSC Advances.
This journal is © The Royal Society of Chemistry 2020

Electronic Supporting Information

DOI: 10.1002/ppsc. ((please add manuscript number))
Article type: Full Paper

Cost-effective fabrication of photopolymer molds with multi-level microstructures for PDMS microfluidic devices manufacture

Carol M. Olmos, Ana Peñaherrera, Gustavo Rosero, Karla Vizuete, Darío Ruarte, Marie Follo, Andrea Vaca, Carlos R. Arroyo, Alexis Debut, Luis Cumbal, Maximiliano S. Pérez, Betiana Lerner, Roland Mertelsmann.

Fmold fabrication

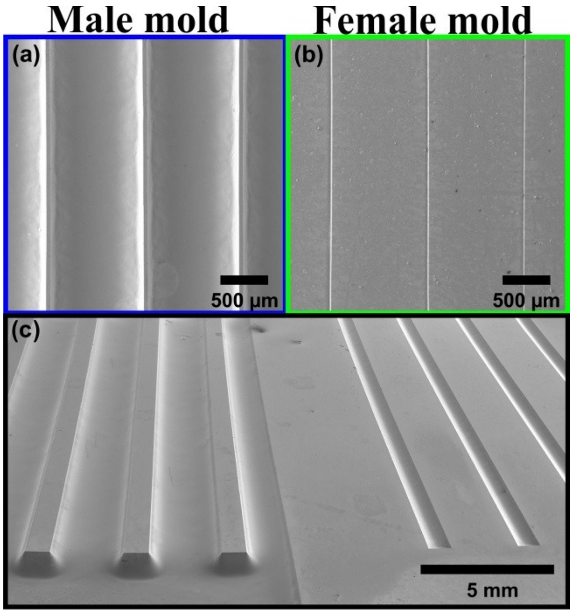

Figure S1. Photopolymer molds from Flexcel NX. (a)Positive structures, (b) Negative structures, (c) Single photopolymer mold with positive and negative structures.

Cell Culture

Jurkat cells Culture

Figure S2 presents a collection of images of the 11 wells of the first line of the microfluidic device.

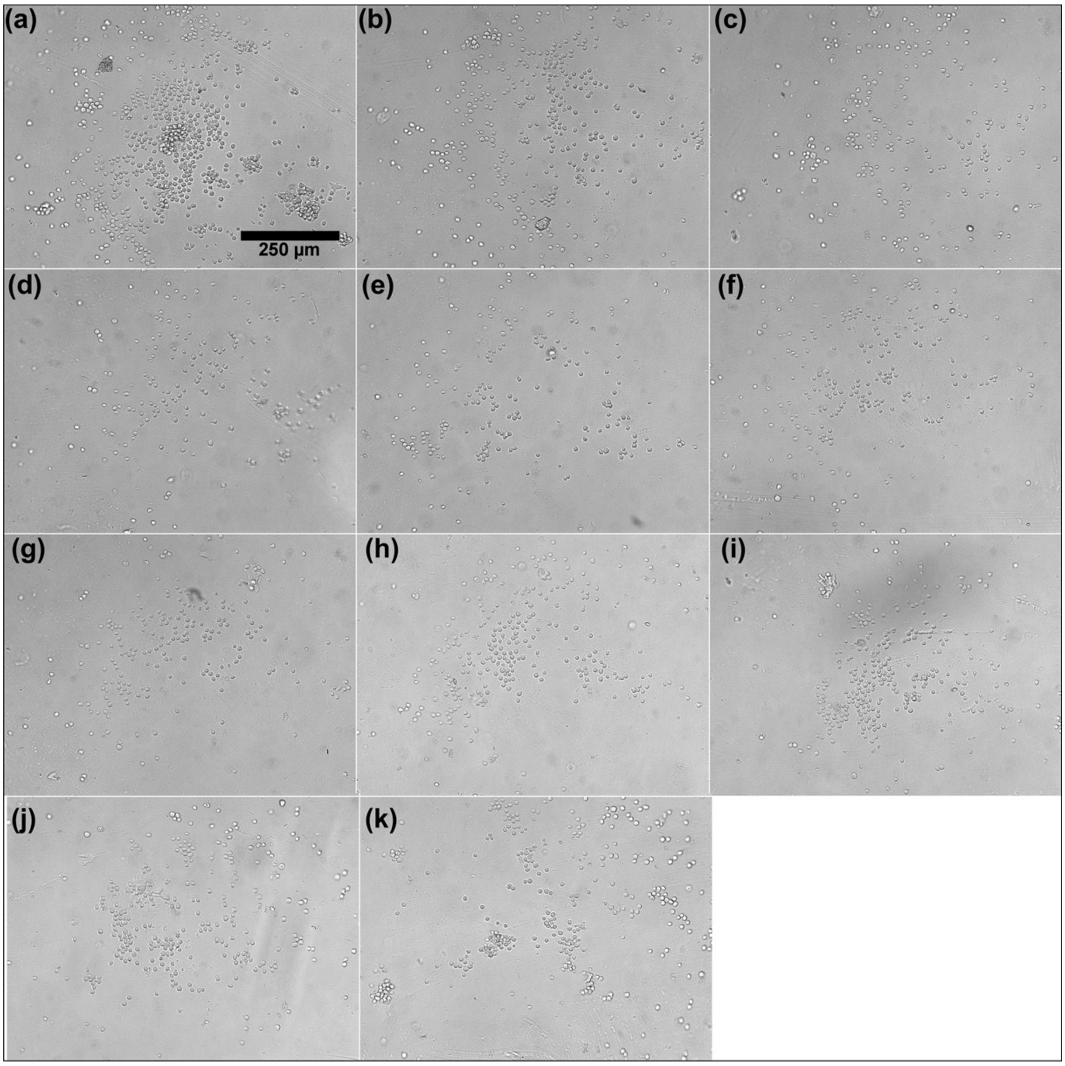

Figure S2. Initial distribution on Jurkat cells cultured in the microfluidic device. Images of eleven wells are shown. (a) to (k) correspond to L1_W1 to L1_W11, respectively**.**

Quantification of the area occupied by cells

Image scale setup.

The image spatial scale (pixel-pixel) was acquired from the Zeiss Inverted fluorescence microscope (Zeiss AxioObserver.Z1 / Apotome, Lighhouse Core Facility Freiburg), with climate chamber and motorized stage. The scale corresponds to 0.65µm = 1 pixel.

Images refining and area quantification.

To perform an automated method to quantitate cells per image, a macro written in FIJI ImageJ platform (version 1.49, http://imagej.nih.gov/ij/; provided in the public domain by the National Institutes of Health, Bethesda, MD, USA) was applied to estimate the area occupied by suspension cells in each image[1]. First, the contrast was enhanced and a band pass filter was applied, afterwards convolve tool was utilized to increase image resolution and brightness to quantify cells. Then, auto threshold was set to create a mask and measure tool was applied to report the percentage of covered area.

Video

Video S1

After cell seeding, one of the wells of the microfluidic device was imaged with a LUCPLFLN 20x/0.45 objective with an Olympus Scan^R High Content Screening Station with a climate chamber set at 37 °C and 5 % CO_2 to obtain an image every 2 minutes over a period of 7 hours. The image sequence shown in Video S1 presents Jurkat cells falling and crawling to the bottom of the well.

References

1 J. Schindelin, I. Arganda-Carreras, E. Frise, V. Kaynig, M. Longair, T. Pietzsch, S. Preibisch, C. Rueden, S. Saalfeld, B. Schmid, J. Y. Tinevez, D. J. White, V. Hartenstein, K. Eliceiri, P. Tomancak and A. Cardona, *Nat. Methods*, 2012, 9, 676–682.
